# Enhancing CDC and ADCC of CD19 Antibodies by Combining Fc Protein-Engineering with Fc Glyco-Engineering

**DOI:** 10.3390/antib9040063

**Published:** 2020-11-17

**Authors:** Sophia Roßkopf, Klara Marie Eichholz, Dorothee Winterberg, Katarina Julia Diemer, Sebastian Lutz, Ira Alexandra Münnich, Katja Klausz, Thies Rösner, Thomas Valerius, Denis Martin Schewe, Andreas Humpe, Martin Gramatzki, Matthias Peipp, Christian Kellner

**Affiliations:** 1 Division of Stem Cell Transplantation and Immunotherapy, Department of Medicine II Christian-Albrechts-University Kiel and University Hospital Schleswig-Holstein, Campus Kiel, 24105 Kiel, Germany; sophia.rosskopf@onlinehome.de (S.R.); klara.eichholz@gmx.de (K.M.E.); katarina_julia@icloud.com (K.J.D.); k.klausz@med2.uni-kiel.de (K.K.); t.roesner@med2.uni-kiel.de (T.R.); t.valerius@med2.uni-kiel.de (T.V.); m.gramatzki@med2.uni-kiel.de (M.G.); 2 Pediatric Hematology/Oncology, Christian-Albrechts-University Kiel and University Hospital Schleswig-Holstein, Campus Kiel, 24105 Kiel, Germany; Dorothee.Winterberg@uksh.de (D.W.); Denis.Schewe@uksh.de (D.M.S.); 3 Department of Transfusion Medicine, Cell Therapeutics and Hemostaseology, University Hospital, LMU Munich, 81377 Munich, Germany; Sebastian.Lutz@med.uni-muenchen.de (S.L.); Ira.Muennich@med.uni-muenchen.de (I.A.M.); Andreas.Humpe@med.uni-muenchen.de (A.H.); Christian.Kellner@med.uni-muenchen.de (C.K.)

**Keywords:** antibody therapy, cluster of differentiation 19 (CD19), CD19, Fc fragment crystallizable (Fc), Fc engineering, complement-dependent cytotoxicity (CDC), antibody-dependent cell-mediated cytotoxicity (ADCC)

## Abstract

Background: Native cluster of differentiation (CD) 19 targeting antibodies are poorly effective in triggering antibody-dependent cell-mediated cytotoxicity (ADCC) and complement-dependent cytotoxicity (CDC), which are crucial effector functions of therapeutic antibodies in cancer immunotherapy. Both functions can be enhanced by engineering the antibody’s Fc region by altering the amino acid sequence (Fc protein-engineering) or the Fc-linked glycan (Fc glyco-engineering). We hypothesized that combining Fc glyco-engineering with Fc protein-engineering will rescue ADCC and CDC in CD19 antibodies. Results: Four versions of a CD19 antibody based on tafasitamab’s V-regions were generated: a native IgG1, an Fc protein-engineered version with amino acid exchanges S267E/H268F/S324T/G236A/I332E (EFTAE modification) to enhance CDC, and afucosylated, Fc glyco-engineered versions of both to promote ADCC. Irrespective of fucosylation, antibodies carrying the EFTAE modification had enhanced C1q binding and were superior in inducing CDC. In contrast, afucosylated versions exerted an enhanced affinity to Fcγ receptor IIIA and had increased ADCC activity. Of note, the double-engineered antibody harboring the EFTAE modification and lacking fucose triggered both CDC and ADCC more efficiently. Conclusions: Fc glyco-engineering and protein-engineering could be combined to enhance ADCC and CDC in CD19 antibodies and may allow the generation of antibodies with higher therapeutic efficacy by promoting two key functions simultaneously.

## 1. Introduction

Therapeutic antibodies have considerably improved treatment outcomes both in solid tumors and in hematological malignancies [1]. In the treatment of lymphomas, antibody therapy is well established and both native antibodies such as rituximab and immunoconjugates have been approved for clinical use. Besides cluster of differentiation (CD) 20, the CD19 antigen represents an attractive target for antibody-based immunotherapy of B-lineage lymphomas and leukemias [2,3]. CD19 shows a favorable expression pattern, since its expression is restricted to the B-cell lineage, where it is displayed from very early to mature stages of B cell differentiation. However, the clinical development of CD19 antibodies was hampered by a lack of efficacy of native IgG1 antibodies. Thus, in contrast to CD20 antibodies, native CD19 antibodies are unable to elicit antibody key effector functions, since they do not induce growth arrest or programmed cell death and are only poorly effective in triggering complement-dependent cytotoxicity (CDC), antibody-dependent cell-mediated cytotoxicity (ADCC) or antibody-dependent cellular phagocytosis (ADCP). Strategies to target CD19 mainly focused on T cell recruitment [4], which led to clinical approval of the [CD19 × CD3] bispecific T cell engager (BiTE) molecule blinatumomab and two chimeric antigen receptor (CAR) T cell products, tisagenlecleucel and axicabtagen–ciloleucel [5,6]. However, most recently, the CD19 antibody tafasitamab (formerly MOR208 or Xmab^®^5574), which was optimized by engineering its fragment crystalizable (Fc) domain to overcome limitations of native CD19 antibodies, has demonstrated clinical efficacy and has received approval by the FDA for combination treatment with the immunomodulatory drug lenalidomide in diffuse large B cell lymphoma (DLBCL) patients [7,8].

Key observations underlining the importance of antibody functions that depend on the Fc domain such as CDC or the recruitment of effector cells for ADCC by engagement of Fcγ receptors (FcγR) on various effector cells have provided a rational basis for the development of Fc engineering strategies for the generation of tailor-made antibodies with enhanced efficacy [9]. The importance of CDC has been demonstrated in selected murine xenograft models [10] and clinical observations have suggested a role for CDC in CD20 antibody therapy. Thus, the consumption of complement proteins following rituximab injection has been observed in lymphoma patients and individual patients benefited from the administration of plasma as a complement source [11,12]. In addition, augmented expression of the inhibitory membrane-bound complement regulatory protein (mCRP) CD59 has been related to rituximab resistance in chronic lymphocytic leukemia (CLL) patients [13]. Besides its potential contribution to the therapeutic activity of monoclonal antibodies, complement activation has also been associated with first infusion reactions.

The importance of effector cell recruitment was demonstrated in murine xenograft models [14,15]. Moreover, clinical observations suggest the importance of effective FcγR engagement also in patients. Thus, lymphoma patients homozygous for the FcγRIIIA-158V allelic version, which is bound by the antibody’s Fc region with higher affinity, showed better responses to rituximab therapy than did patients carrying the low-affinity FcγRIIIA-158F allele, suggesting functions as ADCC or ADCP as important mechanisms by which the antibody depletes lymphoma cells [16,17,18]. However, a consistent effect of FcγR genotype on the clinical anti-tumour activity of therapeutic IgG1 antibodies has not been observed in all published clinical studies [19,20].

Currently, two main Fc engineering technologies exist, which either rely on modifying the Fc-associated glycan linked to amino acid N297 or on altering the amino acid sequence in the C1q and FcγR binding sites within the antibody constant heavy chain 2 (CH2) domain [9]. For example, the fucose content in antibody preparations was reduced and afucosylated antibodies or antibodies with significantly reduced fucose content exerted a higher affinity to FcγRIII (CD16), whose activating isoform (FcγRIIIA) is expressed by natural killer (NK) cells, macrophages and certain γδ-T cell subsets in humans, while binding to other FcγR was not affected. Alternatively, Fc protein-engineering was shown to be a valid approach to improve Fc mediated antibody functions. Amino acid substitutions were identified that greatly improved binding to activating FcγR and enhanced the antibody’s ability to trigger NK cell ADCC or ADCP by macrophages. Other substitutions were demonstrated to enhance CDC by improving binding to C1q [21,22]. However, maintenance of ADCC function was difficult in such engineered antibodies optimized for C1q binding, because certain modifications that on the one side enhanced CDC diminished on the other side FcγR binding and ADCC. Therefore, additional amino acid substitutions were necessary. For example, a gain in CDC was achieved by the introduction of amino acid exchanges S267E/H268F/S324T in the CH2 domain, but the two additional substitutions G236A/I332E were also necessary to preserve ADCC activity (“EFTAE modification”) [21]. Moreover, mixed isotype IgG1/IgG3 antibodies exerted improved CDC activity, and also the introduction of certain amino acid exchanges that promote assembly of antibody hexamers augmented CDC [23]. CDC was further improved by combinations of such antibodies recognizing different antigens such as CD20 and CD37 that are co-expressed on certain lymphomas [24]. Yet, simultaneous enhancement of both ADCC and CDC functions to increase the potency of native IgG1 molecules by amino acid alteration was difficult, presumably because the binding sites for FcγR and C1q overlap [25,26,27].

Fc engineering technologies are particularly important for improving CD19 antibodies that in general exert poor effector functions [4]. Thus, Fc engineering has been applied for CD19 antibodies to favor effector cell recruitment and resulted in CD19 antibodies now being capable of triggering ADCC and ADCP effectively. Thus, CD19 antibodies carrying amino acid substitutions S239D/I332E (“DE modification”) such as antibody tafasitamab (formerly MOR208 or Xmab^®^5574) were found to be more effective in inducing NK-cell-mediated ADCC and ADCP by macrophages [7,28,29]. Importantly, the comparison of tafasitamab with its native counterpart revealed that in non-human primates Fc engineering was essential for B cell depletion [30]. Clinically, promising results were obtained with tafasitamab single-agent therapy in B cell Non-Hodgkin lymphoma [31], and therapeutic efficacy has been demonstrated for this antibody in combination with lenalidomide in DLBCL not eligible for autologous stem-cell transplantation [8]. Recently, tafasitamab in combination with lenalidomide has received approval by the FDA for the treatment of adult patients with relapsed or refractory DLBCL, making the antibody the fourth clinically approved Fc engineered antibody optimized for enhanced FcγR binding in oncology next to mogamulizumab, obinutuzumab and Belantamab–Mafodotin, which bind the CC chemokine receptor 4, CD20 and B cell maturation antigen (BCMA), respectively [32]. Besides, Fc glyco-engineered, afucosylated CD19 antibodies demonstrated enhanced efficacy in triggering ADCC or ADCP and exerted therapeutic efficacy in pre-clinical models [33,34]. Clinically, promising results were obtained for monotherapy with the CD19 antibody inebilizumab, which is approved for treatment of the autoimmune disease neuromyelitis optica spectrum disorder, in a phase I study in relapsed or refractory lymphoma patients [35,36]. Finally, the feasibility to enhance CDC activity of CD19 antibodies by Fc engineering has been demonstrated by introducing the EFTAE amino acid modifications to optimize C1q binding, resulting in a CD19 antibody with potent CDC function [21]. However, Fc engineered CD19 antibodies with established CDC and ADCC activity have not been described yet.

Recently, we have shown that ADCC and CDC by CD20 antibodies can be enhanced simultaneously by concomitant Fc glyco- and Fc protein-engineering [37]. Thus, an Fc double-engineered version of rituximab was generated, in which CDC was enhanced by introducing the EFTAE modification, while ADCC was improved by expression of the antibody as an afucosylated variant in Lec13 cells. Here, we investigated whether Fc double engineering was applicable to CD19 antibody using differentially engineered versions based on V-regions of tafasitamab, of which a native IgG1 derivative is ineffective in ADCC and CDC reactions.

## 2. Materials and Methods

### 2.1. Cell Culture

Raji, Ramos, SK-BR-3 (DSMZ—German Collection of Microorganisms and Cell Cultures, Braunschweig, Germany) and baby hamster kidney (BHK)-21 cells (American Type Culture Collection, ATCC, Manassas, VA, USA) were kept in RPMI 1640 Glutamax-I medium (Thermo Fisher Scientific, Waltham, MA, USA) containing 10% fetal calf serum (FCS; Thermo Fisher Scientific, Waltham, MA, USA), 100 U/mL penicillin and 100 µg/mL streptomycin (Thermo Fisher Scientific; R10+ medium). BHK-21 cells that were co-transfected with plasmids encoding the FcεRI γ chain and either human FcγRIIIA 158F (BHK-CD16-158F) or FcγRIIIA 158V (BHK-CD16-158V) were cultured as described [38]. CHO glycosylation mutant Lec13 cells [39,40] were maintained in MEM alpha medium with nucleosides (Thermo Fisher Scientific, Waltham, MA, USA) supplemented with 10% dialyzed FCS (Thermo Fisher Scientific, Waltham, MA, USA) and penicillin (100 U/mL)/streptomycin (100 µg/mL). For culturing CHO-K1 and Lec13 cells transfected with antibody expression vectors, hygromycin B (Thermo Fisher Scientific, Waltham, MA, USA) was added to a concentration of 500 μg/mL. CHO cells stably transfected with a plasmid coding for the cDNA of human CD19 (Origene Technologies Inc., Rockville, MD, USA) were generated using standard procedures (Peipp, unpublished).

### 2.2. Antibodies

For generation of a CD19 antibody variant carrying the EFTAE amino acid modification the variable heavy chain region (VH) of a CD19 antibody (tafasitamab) was excised from vector pSectag2-CD19-HC-DE [28] and cloned as NheI/PpuMI cassette into vector pSectag2-HC-EFTAE [37] encoding a modified human IgG1 Fc region with amino acid modification, harboring the exchanges S267E/H268F/S324T/G236A/I332E [21]. The generation of expression vectors encoding tafasitamab light chain (LC) and a native CD19 IgG1 heavy chain (HC) has been described previously [28]. Fucosylated or non-fucosylated CD19 antibodies were expressed in stably transfected CHO-K1 or Lec13 cells, respectively, and purified by affinity chromatography as described previously [37]. Corresponding control antibodies against HER2 as well as the Fc engineered variant of rituximab CD20-EFTAE-CHO were produced as described earlier [37]. Trastuzumab and rituximab were obtained from Roche (Penzberg, Bavaria, Germany).

### 2.3. Sodium Dodecyl Sulfate Polyacrylamide Gel Electrophoresis (SDS-PAGE), Lectin Blot Analysis, WESTERN Transfer Experiments and Size Exclusion Chromatography (SEC)

Antibody integrity and concentration were determined by reducing or non-reducing SDS-PAGE following published procedures [41]. Lectin blots with biotinylated A. aurantia lectin (Vector Laboratories, Burlingame, CA, USA) and Western Transfer experiments employing goat-anti-human-IgG-HRP conjugates (Sigma Aldrich, St. Louis, MO, USA) for detection of human IgG heavy chains were performed as described [41]. SEC was performed according to standard procedures using an Äkta Pure chromatography system.

### 2.4. Flow Cytometry

Antibody binding to antigen-positive cells was analyzed using secondary Fluorescein isothiocyanate (FITC) or Phycoerythrin (PE) conjugates of anti-human IgG Fc F(ab′)2 fragments of polyclonal goat antibodies (Dianova) and flow cytometry as described [37]. Deposition of C1q was analyzed by incubating 3 × 10^5^ Raji cells with antibodies (25 µg/mL) in 50 µL R10+ medium on ice for 20 min. In parallel, human serum (final concentration of 2%) and antibody eculizumab (200 µg/mL) (Alexion Pharma GmbH; Munich, Germany) were incubated in R10+ medium at room temperature for 20 min to neutralize C5, before 50 µL were reacted with antibody-treated cells. After three washing steps, cell-associated C1q was detected with FITC-conjugated rabbit anti-C1q antibody (DAKO, Glostrup, Denmark) by flow cytometry.

### 2.5. Cytotoxicity Assay

CDC and ADCC were analyzed in 51Cr release assays following published procedures [41]. Mononuclear cells (MNC) and plasma were prepared from citrate-anticoagulated blood from healthy volunteers by density gradient centrifugation employing Easycoll (Biochrom, Berlin, Germany). In CDC experiments, plasma was added to the reactions (25%) as a source of complement and Refludan^®^ (Bayer HealthCare Pharmaceuticals, Wayne, NJ, USA) was used as anticoagulant at concentration of 10 µg/mL. In ADCC experiments, antibodies were analyzed at an effector-to-target cell ratio of 40:1.

### 2.6. Statistical Analysis

Statistical and graphical analyses were performed using software GraphPad Prism 8.0 (GraphPad Software, San Diego, CA, USA). *p*-values were calculated using repeated measures ANOVA and Bonferroni post-tests. Differences between treatment groups were regarded as statistically significant for *p* < 0.05.

## 3. Results

**Figure 1:**
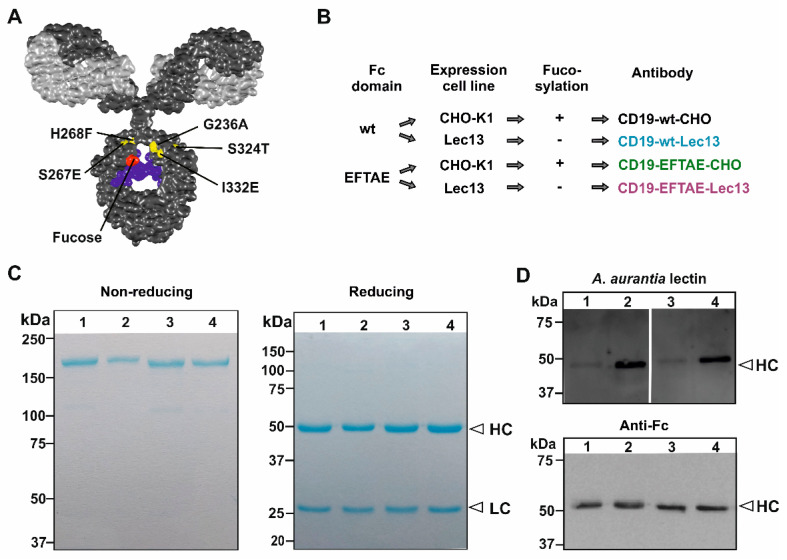
Generation of Fc engineered CD19 antibodies. (**A**) Structural model of an IgG molecule and illustration of amino acid exchanges S267E/H268F/S324T/G236A/I332E (EFTAE modification; in yellow) in the antibody CH2 domain, and the critical fucose residue in red. The light and heavy chains are depicted in light grey and dark grey, respectively. The N297-associated carbohydrate is colored in blue. The model is based on the pdb-file provided by Dr. Mike Clark [42] and was edited employing Discovery Studio Visualizer software (Biovia, San Diego, CA, USA). (**B**) Expression constructs for CD19 heavy chains with native (wt) or with EFTAE modified Fc domain sequences were generated and transfected into CHO-K1 and Lec13 cells for production of fucosylated antibodies (CD19-wt-CHO and CD19-EFTAE-CHO) as well as their afucosylated counterparts (CD19-wt-Lec13 and CD19-EFTAE-Lec13), respectively. (**C**) After purification by affinity chromatography antibodies were analyzed by SDS-PAGE and Coomassie blue staining under non-reducing (left gel) or reducing (right gel) conditions. Amounts of 1–2 µg protein were loaded on 6% and 12% polyacrylamide gels, respectively (Lanes: (1) CD19-EFTAE-Lec13, (2) CD19-EFTAE-CHO, (3) CD19-wt-Lec13, (4) CD19-wt-CHO). Results from one representative experiment are shown (*n* = 3). HC, heavy chain; LC, light chain. (**D**) The fucosylation status of the different antibody versions was determined by lectin blot experiments employing biotinylated A. aurantia lectin and HRP-conjugated neutrAvidin protein (upper panel), indicating that fucose was almost absent in antibodies produced in Lec13 cells. As a control, antibody heavy chains (HC) were detected in Western Transfer experiments with an HRP-coupled anti-human IgG Fc antibody (lower panel). Results from one representative experiment are shown (*n* = 3). Lanes: (1) CD19-EFTAE-Lec13 (2) CD19-EFTAE-CHO (3) CD19-wt-Lec13 (4) CD19-wt-CHO).

**Figure 2:**
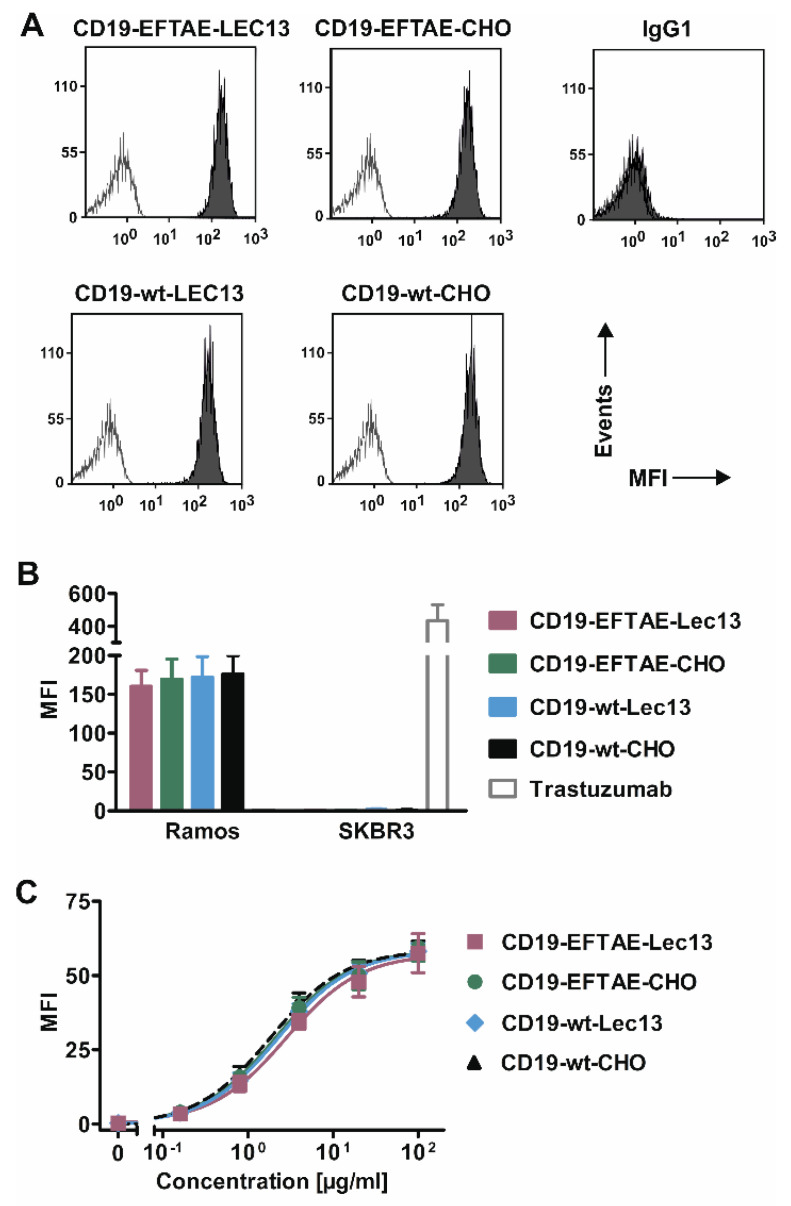
CD19 binding analysis. (**A**) CD19-positive Ramos cells were incubated with antibodies as indicated (concentration: 50 µg/mL; grey peaks) or in PBA buffer alone (white peaks), stained with FITC-coupled anti-human IgG Fc F(ab’)2 and then analyzed by flow cytometry. As a control, trastuzumab was added (IgG1). (**B**) CD19-wt-CHO, CD19-EFTAE-CHO, CD19-wt-Lec13 and CD19-EFTAE-Lec13 (concentration: 50 µg/mL) bound to CD19-expressing Ramos cells but did not react with CD19-negative SK-BR-3 cells. Bars indicate mean values ± SEM (*n* = 3) of mean fluorescence intensity (MFI). PE-labeled anti-human IgG Fc F(ab’)2 fragments were used as secondary antibodies. Trastuzumab was employed as a control antibody and bound to HER2-positive SK-BR-3 cells. (**C**) Binding of antibody versions to CHO-K1-CD19 cells was analyzed at varying concentrations employing FITC-coupled anti-human IgG Fc F(ab’)2 fragments as detection reagents and MFI values were determined by flow cytometry. Mean values ± SEM are shown (*n* = 4).

In an effort to equip CD19 antibodies with both CDC and ADCC functions, an Fc double-engineered antibody version of the CD19 antibody tafasitamab was generated by applying Fc protein-engineering and Fc glyco-engineering technologies (Figure 1A). First, the amino acid substitutions S267E/H268F/S324T/G236A/I332E (EFTAE) were introduced to establish CDC activity [21]. Second, the antibody was produced as an afucosylated variant by expression in Lec13 cells to also enhance its ability to trigger ADCC in parallel. In addition to this double-engineered CD19 antibody referred to as CD19-EFTAE-Lec13, also a native IgG1 version (CD19-wt-CHO) and corresponding mono-engineered variants, i.e., the fucosylated variant with the EFTAE modification (CD19-EFTAE-CHO) and the afucosylated antibody with native IgG1 Fc (CD19-wt-Lec13) were produced using CHO-K1 or Lec13 cells as expression hosts, respectively (Figure 1B). The antibodies were purified from cell culture supernatant by affinity chromatography of established monoclonal production lines, and the integrity of purified antibodies was verified by SDS-PAGE under reducing or non-reducing conditions and Coomassie Blue staining (Figure 1C). Selected variants were analyzed by SEC to investigate the content of multimers/aggregates (Supplementary Figure S1) Analysis of fucosylation status by lectin blot employing A. aurantia lectin revealed that antibodies produced in CHO-K1 cells were fucosylated, while fucose was almost absent in the Fc domain of antibody versions expressed in Lec13 cells (Figure 1D). Binding studies using flow cytometry indicated that all CD19 antibody variants bound to CD19-positive Ramos cells (Figure 2A) and did not react with CD19-negative SK-BR-3 breast cancer cells used as control (Figure 2B). Importantly, the four antibodies showed similar binding to CD19-transfected CHO-K1 cells and exerted almost equal affinity to the target antigen (Figure 2C). EC50 values for binding were between 2 µg/mL (13 nM) and 3 µg/mL (20 nM) for the different CD19 antibodies, in agreement with results obtained for the CD19 antibody variant with DE modification [28].

**Figure 3:**
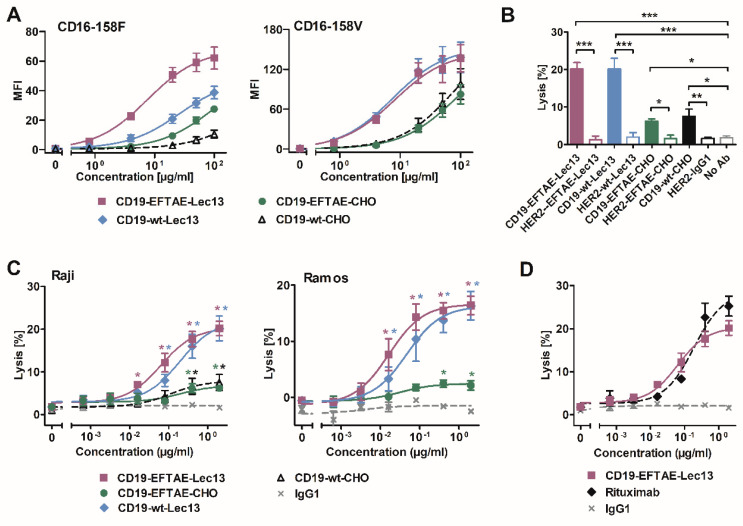
FcγRIIIA binding and induction of ADCC by differentially engineered CD19 antibodies. (**A**) Binding of antibodies CD19-wt-CHO, CD19-EFTAE-CHO, CD19-wt-Lec13 and CD19-EFTAE-Lec13 to transfected BHK cells stably expressing human FcγRIIIA-158V (BHK-CD16-158V) or FcγRIIIA-158F (BHK-CD16-158F) alleles was analyzed by flow cytometry. Secondary FITC-coupled anti-human IgG Fc F(ab’)2 fragments were employed for detection. MFI, mean fluorescence intensity. (**B**) Induction of ADCC by antibody versions (concentration: 2 µg/mL) was investigated in 51Cr release experiments using Raji as target cells and human MNC as effector cells. Similarly designed variants of trastuzumab were used as controls. Bars represent mean values of specific lysis ± SEM. Significant differences between CD19 antibodies and HER2-specific control antibodies or the control reaction performed in the absence of any added antibody (no Ab) are indicated (*, *p* ≤ 0.05; **, *p* ≤ 0.01; ***, *p* ≤ 0.001 *n* = 3). (**C**) Dose-dependent induction of ADCC by CD19 variants was analyzed using Raji (*n* = 3) or Ramos cells as targets and MNC as effector cells. Data points indicate mean values of specific lysis ± SEM. Statistically significant differences in ADCC between CD19 antibodies and the control antibody trastuzumab (IgG1) are indicated (*, *p* ≤ 0.05; **, *p* ≤ 0.01; *n* = 3). (**D**) Comparison of ADCC by the Fc double-engineered antibody CD19-EFTAE-Lec13 (purple) and by the CD20 antibody rituximab (black). Trastuzumab served as an additional negative control (IgG1). Raji cells were used as target cells and MNC served as effector cells. Mean values of specific lysis ± SEM are shown (*n* = 3).

To analyze the impact of fucosylation on FcγRIIIA engagement, dose-dependent binding of the CD19 antibody variants to BHK cells transfected with expression vectors encoding either FcγRIIIA-158V or FcγRIIIA-158F expression constructs was analyzed (Figure 3A). Here, antibody variants differed considerably in their binding affinity. Of note, afucosylated antibodies bound both FcγRIIIA allelic variants with a significantly higher affinity. Thus, Fc glyco-engineering improved binding of both the antibody variant with native amino acid sequence and the version carrying the EFTAE modification. A comparison between the two afucosylated antibodies revealed that whereas they had equal binding to the high-affinity FcγRIIIA-158V allele (EC50 = 50 nM), the double-engineered antibody CD19-EFTAE-Lec13 was superior to CD19-wt-Lec13 in binding to the low-affinity FcγRIIIA-158F allele. Thus, FcγRIIIA-158V transfected cells were bound by CD19-EFTAE-Lec13 as effectively as FcγRIIIA-158F transfected cells (EC50 = 50 nM), whereas CD19-wt-Lec13 bound with lower affinity to FcγRIIIA-158F (EC50 = 180 nM). A benefit of the EFTAE modification was also observed for the fucosylated antibodies, since also CD19-EFTAE-CHO showed better binding than CD19-wt-CHO when FcγRIIIA-158F transfected cells were analyzed (Figure 3A).

To determine the abilities of the antibodies to trigger ADCC, 51Cr release experiments with MNC effector cells and Raji lymphoma target cells were performed (Figure 3B). At saturating conditions, CD19-wt-Lec13 and CD19-EFTAE-Lec13 showed an enhanced potency relative to fucosylated antibodies CD19-EFTAE-CHO and CD19-wt-CHO, which both induced only moderate ADCC. None of the corresponding control antibodies against HER2, which is not expressed by Raji cells, induced ADCC, showing the antigen-specific mode of action even when the antibodies had been Fc engineered. Analysis of dose-dependent ADCC induction using MNC and either Raji or Ramos target cells revealed that CD19-EFTAE-Lec13 and CD19-wt-Lec13 had similar efficacy, although the double-engineered antibody was slightly more effective (Figure 3C). In experiments with Raji cells, EC50 values were 0.4 nM and 1.3 nM for CD19-EFTAE-Lec13 and CD19-wt-Lec13, respectively. However, these differences did not reach statistical significance. A comparison between rituximab and CD19-EFTAE-Lec13 revealed that this antibody now almost reached the potency of rituximab, although rituximab was slightly more effective in terms of maximum lysis at saturating concentrations (Figure 3D). Thus, Fc glyco-engineering by the generation of afucosylated antibodies improved the ADCC of CD19 antibodies, and the inclusion of the EFTAE modification in the afucosylated CD19 antibody even improved ADCC slightly further.

**Figure 4:**
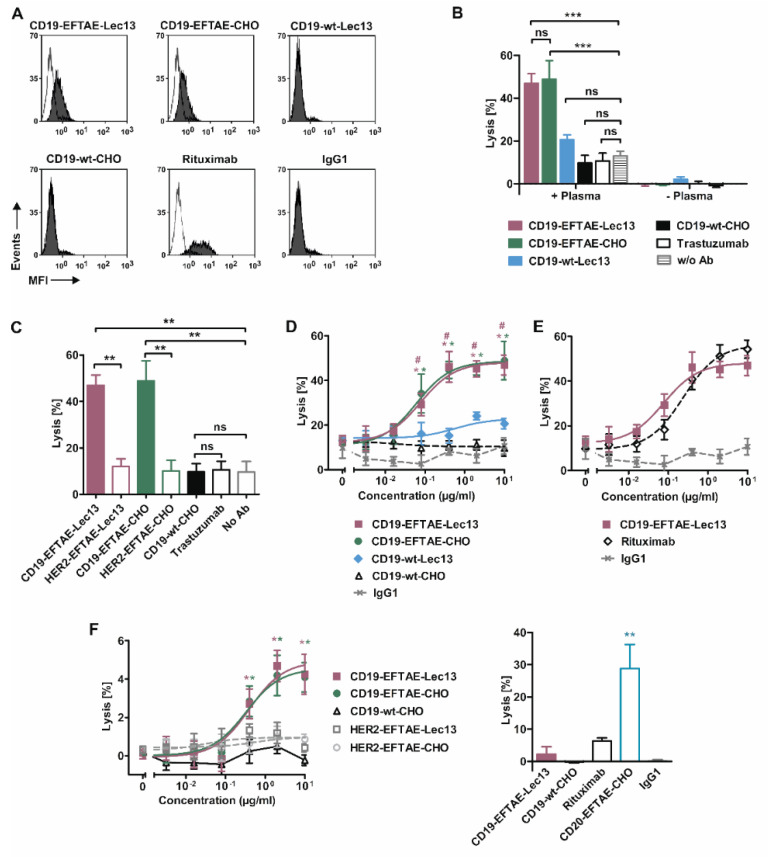
C1q binding capacities and induction of CDC by CD19 antibody versions. (**A**) Raji cells were left untreated (white peaks) or coated with antibodies CD19-wt-CHO, CD19-EFTAE-CHO, CD19-wt-Lec13 or CD19-EFTAE-Lec13 (grey peaks) at a concentration of 50 µg/mL. Then cells were incubated with human serum (1%) as a source of C1q and C1q binding to antibody coated cells was determined using a FITC-conjugated mouse anti-human C1q antibody and flow cytometry. Rituximab, which binds C1q efficiently, and trastuzumab, which does not react with HER2-negative Raji cells, were included as control reagents. MFI, mean fluorescence intensity. (**B**) CDC by CD19 antibodies was determined by 51Cr release experiments with Ramos cells as target cells in the presence or in the absence of 25% human plasma. Antibodies were analyzed at a concentration of 10 µg/mL. Bars represent mean values of specific lysis ± SEM. Significant differences between antibody-treated groups and the control group without any added antibody (w/o Ab) are indicated (***, *p* ≤ 0.001; ns, not significant; *n* = 3). (**C**) CDC against Ramos by antibodies CD19-EFTAE-Lec13, CD19-EFTAE-CHO and CD19-wt-CHO compared to corresponding engineered control antibodies against HER2 and the native anti-HER2 IgG1 antibody trastuzumab. Antibodies were analyzed at a concentration of 10 µg/mL. Bars show mean values of specific lysis ± SEM. Significant differences between CD19 antibodies and the corresponding versions of the HER2-specific antibody trastuzumab or between antibody treatment and the control reaction without any added antibody (no Ab) are indicated (**, *p* ≤ 0.01; *n* = 3). (**D**) Dose-dependent induction of CDC against Ramos cells (*n* = 3). Human plasma (25%) was added as a source of complement. *, statistically significant differences (*p* ≤ 0.05) in CDC between CD19 antibodies and the native CD19-wt-CHO IgG1 molecule; #, statistically significant differences (*p* ≤ 0.05) between CD19-EFTAE-Lec13 and CD19-wt-Lec13. Trastuzumab served as an additional negative control (IgG1). (**E**) Comparison of CDC induced by the Fc double-engineered antibody CD19-EFTAE-Lec13 and by the CD20 antibody rituximab. Trastuzumab served as an additional negative control (IgG1). Ramos cells were employed as target cells and serum was added to 25% as a source for complement. Mean values of specific lysis ± SEM are shown (*n* = 3). (**F**) Left graph: CD19 antibody variants were analyzed at varying concentrations for their ability to induce CDC against Raji cells, which in comparison to Ramos cells are rather resistant to CDC. Mean values of specific lysis ± SEM are shown (*n* = 3). Right graph: CD19 antibody variants were compared with rituximab and an Fc engineered version of rituximab-containing the EFTAE modification (CD20-EFTAE-CHO). Trastuzumab served as an additional negative control (IgG1). Mean values of specific lysis ± SEM are shown and statistically significant differences are indicated (**, *p* < 0.01).

Since the induction of CDC along the classical pathway requires efficient C1q deposition, we investigated whether the EFTAE amino acid substitutions in engineered CD19 antibodies promoted C1q fixation on lymphoma cells and whether this was affected by the antibody fucosylation status (Figure 4A). To test this, CD19-expressing Raji cells were first incubated in the presence of antibodies CD19-wt-CHO, CD19-EFTAE-CHO, CD19-wt-Lec13 or CD19-EFTAE-Lec13. Then cells were reacted with human serum as a source of C1q, which finally was detected using an antibody specific for human C1q. Analysis by flow cytometry demonstrated that cell-bound C1q was only detectable when Raji cells were pre-incubated with CD19 antibody variants carrying the EFTAE modification. Of note, CD19-EFTAE-CHO and CD19-EFTAE-Lec13 were similarly effective in binding C1q, but none of them reached the efficacy of rituximab (Figure 4A).

To investigate CDC induction by CD19 antibodies, 51Cr release assays were performed employing human plasma and CDC-sensitive Ramos cells (Figure 4B). As a result, only antibodies CD19-EFTAE-CHO and CD19-EFTAE-Lec13 were able to trigger efficient CDC, while antibodies CD19-wt-CHO and CD19-wt-Lec13 were not effective. No lysis occurred in the absence of plasma, indicating that under these experimental conditions no direct induction of cell death was induced. Additionally, no CDC was found when HER2-specific control antibodies were applied, revealing that the observed CDC was induced in a target antigen-dependent manner (Figure 4C). Importantly, the analysis of dose-dependent induction of CDC indicated that CD19-EFTAE-Lec13 was as effective as CD19-EFTAE-CHO (Figure 4D). Both antibodies triggered CDC at nanomolar concentrations with EC50 values of 0.5 nM and 0.4 nM for CD19-EFTAE-Lec13 and CD19-EFTAE-CHO, respectively. The Fc double-engineered antibody CD19-EFTAE-Lec13 almost reached the potency of rituximab (Figure 4E). Finally, CDC was analyzed with Raji cells that are rather resistant to CDC (Figure 4F). Both CD19-EFTAE-Lec13 and CD19-EFTAE-CHO were able to trigger CDC against Raji cells in a dose-dependent manner. However, lysis of Raji cells was quite low. Reduced CDC induction was also observed for rituximab, which was employed for comparison (Figure 4F). However, an Fc-engineered variant of rituximab carrying the EFTAE modification (CD20-EFTAE-CHO) [37] was able to trigger substantial CDC, showing that although Fc engineering improves CDC of CD19 antibodies leading to a considerable efficacy, limitations associated with unfavorable antigen characteristics or specific antibody features are not fully overcome, when CDC insensitive target cells are analyzed.

## 4. Discussion

The CD19 antigen has attractive features for antibody therapy of B-cell lineage leukemias and lymphomas, but native CD19 IgG1 isotype antibodies only poorly mediate CDC and ADCC. In an effort to enhance both functions concomitantly, Fc protein-engineering was combined with Fc glyco-engineering to generate a double-engineered version of a CD19 antibody based on the v-regions of the clinically approved antibody tafasitamab. We found that the double-engineered afucosylated CD19 antibody harboring the EFTAE modifications was more efficacious in triggering both ADCC and CDC than the native IgG1 molecule, which had only weak effects in ADCC and which was unable to induce CDC. These findings demonstrate that CDC and ADCC functions can be established in CD19 antibodies by combined glyco-engineering and protein-engineering technologies and show that these technologies are applicable to the same antibody molecule.

The underlying reasons why native CD19 antibodies are not efficacious as for example CD20 antibodies are not fully understood and presumably are not due to antigen expression levels. Potential reasons may be specific antigen characteristics such as antigen membrane fluidity, size and structure or the antigen′s plasma membrane microdomain localization, as well as antibody characteristics such as the epitope specificity and its location [43]. However, even when native antibodies elicit weak effects, they can be turned into effective agents by applying Fc engineering technologies, which allow fine-tuning of individual antibody effector functions and the generation of tailor-made antibodies [9,32].

Regarding CD19, the clinically approved Fc protein engineered antibody tafasitamab with amino acid substitutions S239D/I332E has demonstrated promising results in clinical studies. However, the antibody is optimized for FcγR binding and still lacks CDC activity [29]. Several observations suggest that CDC activity is an important antibody function and establishing CDC activity in CD19 antibodies may be beneficial in certain situations. Thus, in murine tumor models, variation in the relative contribution of CDC and FcγR-mediated functions were observed and an impact of tumor burden and anatomic localization has been suggested [44]. Additionally, the immune status of the patient and the tumor microenvironment may play a role [45]. Moreover, different phenotypes of tumor cells may impact the susceptibility of tumor cells to different antibody functions differentially, and cell phenotypes of individual tumor cells may differ even in the same patient. Thus, susceptibility to ADCC may be hampered by strong expression of inhibitory human leukocyte antigen (HLA) molecules or promoted by increased expression of NK cell-activating danger signals such as NKG2D ligands [46]. In contrast, tumor cells may be protected from CDC by expression of mCRP [47]. Interestingly, studies with CD20 transgenic cell clones revealed that individual CDC resistant cell clones were eliminated by ADCC and vice versa [48]. Thus, CDC as well as effector cell-mediated killing may be required for effective eradication of tumor cells in certain situations, and Fc double-engineered CD19 antibodies optimized for ADCC and CDC activity may be advantageous. Whether the double engineering strategy demonstrated here can be applied also to other CD19 antibodies remains to be investigated.

Of note, Fc glyco-engineering by lowering fucose content enhances only FcγRIIIA affinity, while Fc protein-engineering often leads to improved affinity for different activating FcγR [9]. Therefore, Fc protein-engineered antibodies carrying for example the DE modification may have advantages in engaging macrophages that express FcγRI and FcγRIIA next to FcγRIIIA. In addition, the comparison of Fc protein-engineered and Fc glyco-engineered antibody derivatives revealed that antibodies harboring the DE modification had a significantly higher affinity to FcγRIIIA than afucosylated antibodies [41]. However, afucosylated antibodies had an almost equal potency to trigger ADCC by NK cells, suggesting that the gain in affinity achieved by Fc glyco-engineering is sufficient for potent effector cell recruitment and ADCC. However, whether also the Fc double-engineered antibody CD19-EFTAE-Lec13 is as effective in mediating ADCC as a corresponding CD19 antibody with the DE modification needs to be investigated.

In previous studies, we have demonstrated that neutrophil-mediated ADCC is diminished when antibodies engineered for improved FcγRIIIA binding were compared to wildtype IgG [49,50]. This may be less relevant for CD19 antibodies, since CD19 antibodies do not trigger neutrophil-mediated ADCC (unpublished observation). When applied in vivo, the situation might even be more complex, since FcR-positive cells and complement proteins may compete for Fc binding. For example, Wang and colleagues demonstrated that complement binding to the Fc domain of wildtype antibodies diminishes NK cell activation [51]. Addressing this aspect in vivo in preclinical mouse models is challenging since FcR binding and complement activation of the described engineered Fc domains in commonly used xenograft models may not reflect the human situation. While certain protein-engineered Fc variants demonstrate enhanced binding to all mouse FcγR, glyco-engineering of human IgG1 results in a very minor improvement in mouse FcγR binding [7,52]. Even in complex transgenic mouse models engineered to express all human FcγR on the respective murine effector populations the contribution of the complement system might not be adequately reflected and tumor location and tumor burden may have a significant impact on which effector mechanisms contribute to the therapeutic activity in a given situation [15,53,54]. Therefore, the impact of double-engineering could probably ultimately only be tested in non-human primates or clinical trials.

In conclusion, the combination of Fc glyco-engineering and Fc protein-engineering technologies promotes both CDC and ADCC activity in CD19 antibodies simultaneously and allows the generation of CD19 antibodies with appreciable efficacy. Thus, Fc double-engineering may represent an attractive strategy, which may be in particular advantageous for antibodies directed against antigens as CD19, which are not that well-suited as target antigens for antibody therapy as CD20 or CD38. Thus, the Fc double-engineering approach may offer an opportunity to enhance the efficacy of CD19 antibody therapy and deserves further evaluation.

## Supplementary Materials

The following are available online at https://www.mdpi.com/2073-4468/9/4/63/s1, Figure S1: Size exclusion chromatography (SEC) of CD19 antibody variants.
