# Supplementary material for: Enhancing CDC and ADCC of CD19 Antibodies by Combining Fc Protein-Engineering with Fc Glyco-Engineering
Source: Antibodies (Basel). 2020 Nov 17;9(4):63. doi: 10.3390/antib9040063 (PMC7709100; doi:10.3390/antib9040063)
Supplement: Supplementary file 1 [file antibodies-09-00063-s001.pdf]

Suppl. Fig. 1

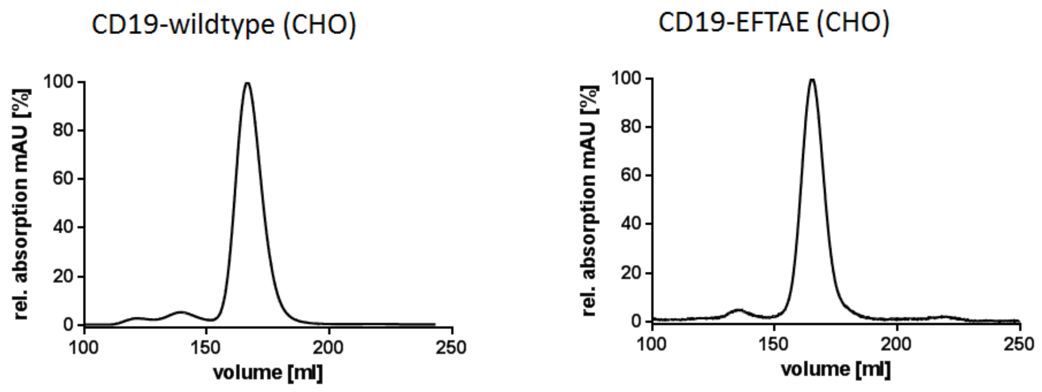

Suppl. Fig.1: Size exclusion chromatography (SEC) of CD19 antibody variants. SEC was performed to demonstrate that introducing the EFTAE amino acid exchanges did not result in an increased level of aggregates. Only minor amounts of aggregates were detected and antibodies harboring wildtype Fc did not significantly differ from antibodies which incorporated the EFTAE amino acid exchanges. Representative experiments are displayed.
